# Research on Earnings Management of Growth Enterprise Market in China Stock Market: Comparative Analysis Based on the BPNN, GBDT, and MLR Models

**DOI:** 10.1155/2022/6064536

**Published:** 2022-05-09

**Authors:** Dian Jia, Ruixiang Xue

**Affiliations:** School of Economics and Management, Qinghai Minzu University, Xining 810007, Qinghai, China

## Abstract

This study, based on 2011–2020 China's listed companies on GEM as research samples, introduces the BPNN (BP neural network) and GBDT (Gradient Boosting Decision Tree) model into the research of the relationship between internal governance and earnings management, which will be comparatively analyzed with the empirical results of the traditional multiple linear regression model, so as to study its validity and predictive power in the earnings' management research field. The results show the following. (1) The matching effect of the multiple linear regression model is poor in the analysis of GEM, with a high rate of experimental data distortion. However, the prediction ability of BPNN and gradient lifting tree model is much better than that of the multiple linear regression model. (2) The gradient lifting tree model is comparatively more suitable for the study of accrual earnings' management, while BP neural network is more suitable for the study of real earnings' management. Through the above research, new ideas will be provided for the application research of machine learning in the future.

## 1. Introduction 

In order to solve the problems of complicated procedures and requirements faced by the newly developing excellent domestic enterprises when they plan IPO, the China Securities Regulatory Commission (CSRC), on June 12, 2020, released “Measures for the Administration of Registration of Initial Public Offerings of GEM (Trial),” “Measures for the Administration of Securities Insurance Registration of GEM (Trial),” “Measures for the Administration of Continuous Supervision of GEM,” and “Measures for the Administration of the Sponsor Business of Securities Issuance and Listing,”

At the same time, the China Securities Regulatory Commission (CSRC), Shenzhen Stock Exchange, China Securities Depository & Clearing Corporation Limited (CSDCC), and the Securities Industry Association issued the related supporting rules, which means that the system of the GEM company issuing securities is officially changed from the approval system to the registration system. The new system was implemented in nationwide pilots. On August 24th, the first registered GEM companies announced IPO in Shenzhen Stock Exchange. For investors, this undoubtedly brings many benefits as well as risks. On the one hand, security issuance audit institutions, according to the registration system, only need to conduct formal examination of registration documents without substantive judgment, which lowers the threshold of listing. On the other hand, most of the GEM-listed companies belong to the emerging industry, facing high market risks and many uncertain factors of performance [1]. It is not rare for some listed company's financial results to become red. The financial risk cannot be overlooked [2]. GEM companies in recent years have frequently suffered chaos [3]. Many enterprises use earnings' management means to whitewash financial statements [4]. Established in October 2009, GEM has been more than ten years old, and its internal governance environment has undergone tremendous changes. Therefore, it is undoubtedly necessary to study earnings' management from the perspective of internal governance.

In the existing research results, however, most scholars' research methods are the multiple linear regression model [5–8]. On the contrary, both the neural network and the gradient lifting tree are common methods that can meet both linear and nonlinear requirements. This paper introduces them to study the relationship between internal governance and earnings management and conducts comparative analyses of the three empirical results to verify the effectiveness and predictive power of the model.

## 2. Materials and Methods

### 2.1. Sample Selection and Data Sources

In this study, companies listed on China's Growth Enterprise Market from October 30, 2011, to December 31, 2020, are selected as research samples. The selection is conducted according to the following criteria. (1) Financial enterprises are excluded. (2) ST and PT listed companies are excluded. (3) Companies of a certain year and a particular industry with sample data less than 10 are excluded. The above continuous variables are treated with 1% of the end. Finally, a total of 2,987 observations of GEM listed companies are obtained in this study, mainly from CSMAR and WIND databases.

### 2.2. Variable Design

#### 2.2.1. Object Variables

*(1) Accrued Earnings' Management*. Accrued earnings' management refers to the purpose of whitewashing financial statements and distorting earnings by corporate executives through using the accounting treatment methods [9]. Many scholars found that the modified Jones model was more suitable for China's capital market [10–11]. The specific model is as follows:

(1) $$\begin{array}{c} \frac{{\text{ETA}}_{t}}{{\text{Asset}}_{\mathrm{t-}1}}=\alpha_{1}\frac{1}{{\text{Asset}}_{\mathrm{t-}1}}+\alpha_{2}\frac{{\Delta \text{REV}}_{t}}{{\text{Asset}}_{\mathrm{t-}1}}+\alpha_{3}\frac{{\text{PPE}}_{t}}{{\text{Asset}}_{\mathrm{t-}1}}+\varepsilon_{t}, \end{array}$$

(2) $$\begin{array}{c} \frac{{\text{NDA}}_{t}}{{\text{Asset}}_{\mathrm{t-}1}}={\overset{\wedge }{\alpha }}_{1}\frac{1}{{\text{Asset}}_{\mathrm{t-}1}}+{\overset{\wedge }{\alpha }}_{2}\frac{{\Delta \text{REV}}_{t}{-\Delta \text{REC}}_{\text{t}}}{{\text{Asset}}_{\mathrm{t-}1}}+{\overset{\wedge }{\alpha }}_{3}\frac{{\text{PPE}}_{t}}{{\text{Asset}}_{\mathrm{t-}1}}, \end{array}$$

(3) $$\begin{array}{c} \text{DA}=\frac{{\text{ETA}}_{t}}{{\text{Asset}}_{\mathrm{t-}1}}-\frac{{\text{NDA}}_{t}}{{\text{Asset}}_{t-1}}. \end{array}$$

In model (1),“ETA” represents the total accrued profit of the company. Its value shows the margin of operating profit and cash flow generated from operating activities. The “Asset” represents the total assets of the company. “△REV” stands for the margin of the operating income of the company between the period *t* and period *t* − 1. “PPE” refers to the fixed assets' net value of the company. “NDA” represents the normal profit of the company, without being manipulated, and “△REC” represents the margin of the company's receivables between the period *t* and period *t* − 1.

The calculation process is as follows. First, the cross-sectional least-squares' regression is done in model (1) to estimate the values of *α*1, *α*2, and *α*3. Then, using model (2), the value of NDAt/Assett-1 can be obtained. The value of ETA/Assett-1 subtracts the absolute value of NDAt/Assett-1 to obtain the degree of earnings' manipulation of the company. The larger the absolute value is, the greater the earnings' manipulation of the company is.

*(2) Real Earnings' Management*. With the gradual improvement of China's capital market, domestic scholars also pay more attention to the real earnings management [12], which refers to the earnings' management activities in which the enterprise manager adjusts and manipulates the openly disclosed accounting earnings by constructing real transaction activities or controlling the occurrence time of related activities. This study adopts the abnormal production cost (PROD), abnormal net operating cash flow (CFO), and abnormal discretionary expense (DISEXP) to construct the company's real earnings' management comprehensive index (REM) [13]. The specific model is as follows:

(4) $$\begin{array}{c} {\text{CFO}}_{t}=\alpha_{\text{0}}+\alpha_{1}\frac{1}{{\text{Asset}}_{t-1}}+\beta_{1}\frac{{\text{REV}}_{t}}{{\text{Asset}}_{t-1}}+\beta_{2}\frac{{\Delta \text{REV}}_{t}}{{\text{Asset}}_{t-1}}+\varepsilon_{t}, \end{array}$$

(5) $$\begin{array}{c} {\text{PROD}}_{t}=\alpha_{\text{0}}+\alpha_{1}\frac{1}{{\text{Asset}}_{t-1}}+\beta_{1}\frac{\text{REV}}{{\text{Asset}}_{t-1}}+\beta_{2}\frac{\Delta \text{REV}}{{\text{Asset}}_{t-1}}+\beta_{3}\frac{{\Delta \text{REV}}_{t-1}}{{\text{Asset}}_{t-1}}+\varepsilon_{t}, \end{array}$$

(6) $$\begin{array}{c} {\text{DISEXP}}_{t}=\alpha_{\text{0}}+\alpha_{1}\frac{1}{{\text{Asset}}_{t-1}}+\alpha_{2}\frac{{\text{REV}}_{t-1}}{{\text{Asset}}_{t-1}}+\varepsilon_{t}, \end{array}$$

(7) $$\begin{array}{c} \text{REM}={\text{PROD}}_{t}-{\text{CFO}}_{t}-{\text{DISEXP}}_{t}. \end{array}$$

#### 2.2.2. Input Variables

*(1) Equity Structure*. “X1” refers to the shareholding ratio of the largest shareholder. As it is known to all, a prominent feature of listed companies in China is the “dominance of one share,” which often leads to the encroachment of major shareholders on the interests of minority shareholders [13]. Therefore, hypothesis1 is proposed. The proportion of the largest shareholder's shareholding is positively related to earnings' management.

“X2” refers to the shareholding ratio of state-owned shares. Since shareholders of state-owned shares have no ownership, they lack sufficient motivation to supervise management so that the management has more opportunities for earnings' management [14]. Therefore, Hypothesis2 is that the proportion of state-owned shares is positively correlated with earnings management.

“X3” refers to the corporate shareholding ratio. Compared with state-owned shares, corporate shares have a higher enthusiasm for the supervision of corporate management. In comparison with minority shareholders, corporate shares have higher supervision ability [15]. Therefore, Hypothesis3 is put forward so that the ratio of corporate shares is negatively correlated with earnings management.

*(2) Board of Directors*. “X4” refers to the chairman of the board is concurrently the general manager if the chairman of the board concurrently holds the position of general manager, which is represented with 1, if not, with 0. The chairman is the head of the board of directors, while the general manager is the head of the company's management. If one person holds the two positions, the supervision of the board of directors of management will undoubtedly be greatly weakened [16]. Therefore, Hypothesis4 is proposed that whether the chairman is concurrently the general manager is positively correlated with earnings' management.

“X5” refers to the proportion of independent directors. The listed company's independent director refers to the director who does not have any position, but is just a director in the listed company, and does not own relationship with the listed company [17]. The listed company's major shareholders may prevent him from making independent and objective judgments. The proportion of independent directors will also affect the company earnings' management. Thus, it can put forward Hypothesis5 that the proportion of independent directors is negatively correlated with earnings management.

“X6” refers to the number of directors. According to the Company Law, a limited liability company shall own a board of directors, 3 to 13 members. If the number of directors is too large, it will cause low efficiency and a high cost of communication between directors, that is, the cost paid will exceed the value created, which will promote earnings' management of the company [18]. Therefore, Hypothesis 6 is put forward that the numbers of the board of directors are positively related with earnings management.

*(3) Board of Supervisors*. “X7” refers to scale of the board of supervisors. The board of supervisors is the supervisory organization of the company [19]. The larger its scale is, the more conducive the supervision of management is. Therefore, Hypothesis 7 is put forward that the size of the board of supervisors is positively correlated with earnings management.

“X8” refers to the shareholding ratio of members of the Board of Supervisors. The purpose of establishing the board of supervisors is to supervise the management of the company [20]. A relatively high shareholding ratio can increase the motivation of the members of the board of supervisors to supervise the operation of the company's management, to realize the suppression of earnings' management behavior. Therefore, Hypothesis 8 is put forward that the size of the board of supervisors is negatively correlated with earnings' management.

*(4) Other Variables*. In view of the fact that the listed companies' degree of earnings' management will inevitably be affected by some nongovernance structure factors and in order to adjust the influence of explanatory variables on explained variables, this study sets the following variables: company size (considering the company's natural logarithm of the total assets at the end of the year), return on equity, and asset-liability ratio [21–23].

### 2.3. Model Construction

#### 2.3.1. Data Normalization

As selected indicators use different dimensions and orders of magnitude, standardization is required to transform them into dimensionless indicators [24]. The calculation process is shown in

(8) $$\begin{array}{c} X*=\frac{X-\text{Min}}{\text{Max}-\text{Min}}, \end{array}$$

where Max represents the maximum of the variable and Min is the minimum.

#### 2.3.2. Multiple Linear Regression

Multiple linear regression is a method to study the relationship between explained variables and explanatory variables. It explores and determines the correlation between variables and the degree of correlation, establishes a regression model, tests the degree of correlation between variables, evaluates and forecasts, and so on [25]. The specific model is as follows:

(9) $$\begin{array}{c} y=\beta_{0}+\beta_{1}•X_{1}+\beta_{2}•X_{2}+,\cdots \cdots ,+\beta_{7}•X_{7}+\varepsilon , \end{array}$$

where the numbers *β*_0_, *β*_1_,…, *β*_*N*_ are unknown irrelevant parameters to *X*_0_, *X*_1_, *X*_2_,…, *X*_*n*_ and “*ε*” is the residual. The multivariate linear regression model has five assumptions. (1) Explanatory variables are uncorrelated; that is, there is no multicollinearity. (2) The random error terms have zero mean and homoscedasticity. (3) The random error terms are no sequence correlation. (4) There is no correlation between random error terms and explanatory variables. (5) The random error terms are normally distributed.

#### 2.3.3. BP Neural Network (BPNN)

Its calculation process consists of the forward calculation process and reverse calculation process. The forward propagation process refers to processing layer by layer from the input layer through the hidden unit layer and then to the output layer. If the desired output cannot be obtained (the threshold of activation function cannot be reached), the forward propagation process is transferred to the back propagation, the weight of each neuron is modified through the optimization algorithm, and the iterative process continues until the threshold is reached, so as to minimize the error signal.

Compared with multiple linear regression, BPNN has the following advantages. Firstly, it does not need to meet the strict assumptions. Secondly, it can satisfy both linear and nonlinear mapping and deal with complex and changeable problems. Therefore, based on the above advantages and considering the complex and diverse relationship between internal governance structure and earnings' management, we try to introduce BPNN into the study.

**Figure 1:**
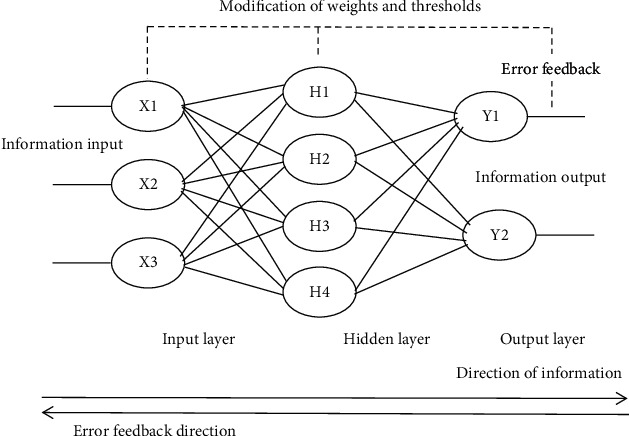
The flowchart of BP neural network.

It can be seen from Figure 1 that the information input variables *X*_1_, *X*_2_,…, *X*_*n*_ are the input variables of the neuron. They are the independent variables which have the key rule on the system model. *W*_1_, *W*_2_,…, *W*_*n*_ are the weight coefficients that adjust each input variables. Many ways can combine and input signals to neurons. The net input of Net in neuron can be obtained by selecting the most convenient linear weighted sum. The specific calculation process is shown in model:

(10) $$\begin{array}{c} {\text{Net}}_{\text{in}}=\sum_{i=1}^{n}w_{i}*x_{i}. \end{array}$$

The letter “*θ*” represents the neuron's threshold value. According to the knowledge of biology, only if the neuron's information reaches the threshold will the neuron be activated. Therefore, the neuronal net input “Netin” and the neuron's threshold value “*θ*” are compared and processed by activation functions to generate the output of neurons. The specific model is as follows:

(11) $$\begin{array}{c} y_{i}=f\left({\text{Net}}_{\text{in}}-\theta \right). \end{array}$$

Because the values of independent variables in this study are not negative and in order to improve the speed and accuracy of training, “Rule” is introduced as the activation function, and the expression is as follows:

(12) $$\begin{array}{c} \text{Relu}=\max\left(0,x\right). \end{array}$$

In order to make the predicted value approximate to the real value and improve the fitting effect, this study adopts the Adam function as the optimization algorithm and constantly iterates to adjust the weight.

#### 2.3.4. GBDT Model

**Figure 2:**
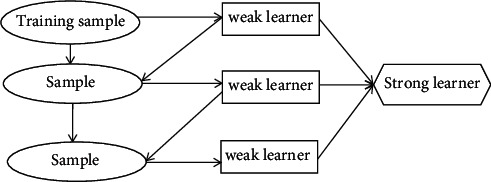
Flowchart of the gradient boosting decision tree.

GBDT firstly uses the training sample for initial training to establish a weak classifier and then calculates the fitting residual error; by learning the error, a new weak classifier will be built. The next weak classifier is actually learning the negative gradient of the former weak classifier's loss function. This circulation will ultimately unite a large number of weak classifiers as a strong classifier, minimizing the loss function to achieve the purpose of forecast, among which the basic learner is regression tree CART, and the general process of GBDT algorithm can be abstracted as Figure 2.

The basic process of constructing the GBDT model is as follows:

(1) Initialize the function model as
  (13) $$\begin{array}{c} F_{0}\left(x\right)=\arg\min\sum_{i=1}^{N}L\left(y_{i},c\right). \end{array}$$
(2) Perform the following actions for each regression tree.
  (a) The gradient value of the loss function of the current model is calculated for each sample as
    (14) $$\begin{array}{c} r_{mi}=-{\left[\frac{\partial L\left(y_{i},F\left(x_{i}\right)\right)}{\partial f\left(x_{i}\right)}\right]}_{f\left(x\right)-f_{m-1}\left(x\right)}. \end{array}$$
  (b) A weak classifier matches a given r_*mi*_, and the leaf node region r_*mj*_ of the number “*M*” tree is calculated (*j* = 1, 2,…, *J*).
  (c) For *j* = 1, 2,…, *J*, the parameter of each leaf node is calculated; the expression is as follows:
    (15) $$\begin{array}{c} c_{mj}=\arg\min\overset{N}{\underset{x_{i}\wedge R_{mj}}{\wedge }}L\left(y_{i},F_{m-1}\left(x_{i}\right)+c\right). \end{array}$$
  (d) Update the model according to different learning rate *η*:
    (16) $$\begin{array}{c} F_{m}\left(x\right)=F_{m-1}\left(x\right)+\eta \overset{J}{\underset{j=1}{\wedge }}c_{mj}I\left(x\wedge R_{mj}\right). \end{array}$$
  (e) To unite a large number of weak classifiers according to different learning rates, the result is a strong classifier. The specific model is as follows:

(17) $$\begin{array}{c} \overset{\wedge }{F}\left(x\right)=F_{M}\left(x\right)=\sum_{m=1}^{M}\eta \sum_{j=1}^{J}c_{mj}I\left(x\in R_{mj}\right). \end{array}$$

## 3. Results

### 3.1. Descriptive Statistics

In order to have an overall understanding of the existing data, descriptive statistics are carried out, and the results are shown in Table 1.

### 3.2. Multiple Linear Regression

**Figure 3:**
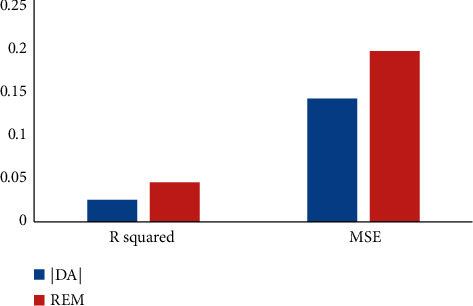
Fitting effect of multiple linear regression.

Figure 3 shows *R*^2^ and MSE of multiple linear regression results. By putting a dependent variable and independent variables in the multivariate linear regression model, it is shown that the multiple linear regression can study both the listed companies' GEM real earnings' management and accrued earnings' management if the value “p” is less than 0.01. From the point of the imitative effect, the fitting effect accrued earnings' management “*R*^2^” is 0.0232, and after being adjusted, “*R*^2^” is 0.0184. The mean square error is 0.14089. Real earnings' management “*R*^2^” is 0.0437, and after being adjusted, “*R*^2^” is 0.039. The mean square error is 0.19652. Thus, it can be seen that the fitting effect is poor and the probability of information distortion is large.

**Figure 4:**
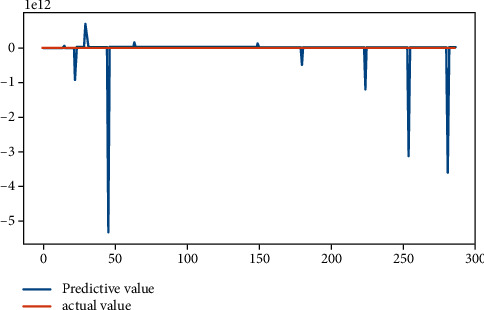
Fitting effect of accrual earnings' management.

**Figure 5:**
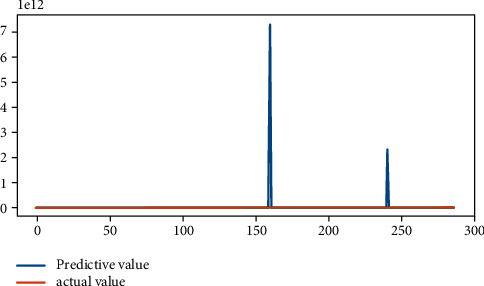
Fitting renderings of real earnings' management.

From Table 2, it can be seen that the results of linear regression analysis. Because the selected indicators' dimension is different in this study, the explained variables “|DA|” and “REM,” according to the precondition of data normalization, are, respectively, treated as explained variables, which are analyzed in the multiple linear regression. The result shows that the return on the equity, the asset-liability ratio, and the accrued earnings' management are positively correlated on the 1% significant level. The largest shareholder's shareholding ratio is negatively related with accrued earnings' management at the significance level of 5%, while the total size of supervisors is positively related with the accrued earnings management at the significance level of 5%. The asset-liability ratio is positively related with real earnings' management at a significant level of 1%, but the company size is negatively correlated. The legal person's shares proportion is positively related with real earnings' management with a significant level of 10%. The fitting effect of linear regression can be seen from Figures 4 and 5.

### 3.3. BP Neural Network

#### 3.3.1. Parameter Setting

To set the input layer and the output layer, there are 11 independent variables in this study, which are used as nodes to input BP neural network. Because there are two target variables in this study, accrual earnings' management and real earnings' management are, respectively, taken as the output layers.

*(1) To set the Hidden Layer*. Choosing the appropriate number of nodes of the hidden layer are of vital importance to the design of BPNN. If the number of nodes of the hidden layer is not enough, the BPNN model will not be able to identify samples, which will affect classification and reduce the accuracy. On the contrary, if the nodes' number in the hidden layer is designed too many, the network structure will be too complex and other problems will occur, such as good training simulation duration and low accuracy, failing to meet the initial setting requirements of the BPNN model, and the problem of overfitting is unavoidable. Therefore, this study refers to relevant literature [13–16] and sets the number of hidden layers as two layers, with 40 layers for each layer.

*(2) To Train Parameter Settings*. In order to improve the fitting effect, this study will be about 90% of all samples as training sets and the remaining 10% as a test sets. The number of iterations will be set up to 1000 times maximally at the same time. The Adam optimization algorithm will be introduced with the “Rule” as the prediction value of the activation function output. To prevent excessive fitting phenomenon, the alpha regularization coefficient is set as 0.01.

#### 3.3.2. Analysis Results

**Figure 6:**
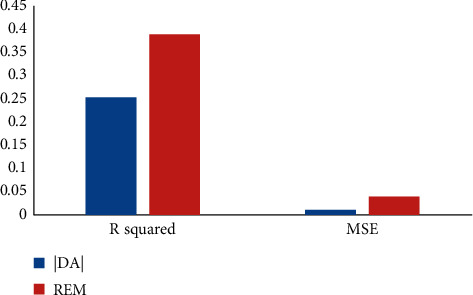
Fitting effect of the neural network.

**Figure 7:**
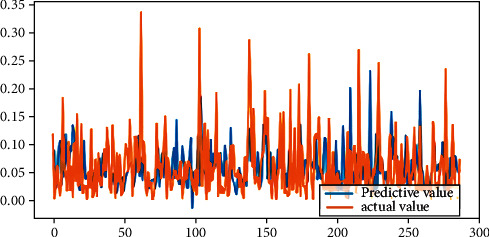
Fitting effect of accrual earnings' management.

**Figure 8:**
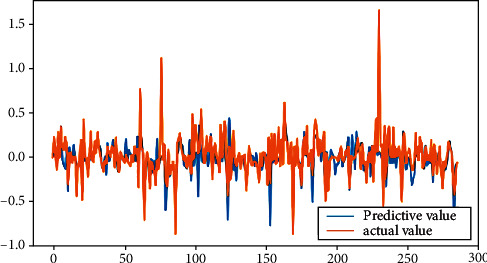
Fitting renderings of real earnings' management.

Figure 6 shows *R*^2^ and MSE of BP neural network results. By setting the parameters into the BPNN, taking the accrued earnings' management and real earnings' management as the target variables, respectively, and comparing the predicted values and the real value, the accrual earnings' management of *R*^2^ is calculated to be 0.2504234, with MSE 0.008057, and the real earnings' management *R*^2^ is 0.3876264, with MSE 0.037542. It shows that the fitting degree of them is higher, but the mean square error (MSE) is lower, which have been largely overlapped. The fitting effect of neural network can be seen from Figures 7 and 8.

### 3.4. Gradient Boosting Decision Tree

#### 3.4.1. Parameter Setting

By referring to the relevant papers [17–20] and considering the particularity of the subject studied in this study, parameter settings are shown in Table 3.

#### 3.4.2. Result Analysis

**Figure 9:**
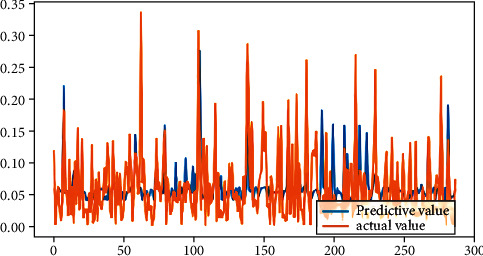
Fitting effect of accrual earnings' management.

**Figure 10:**
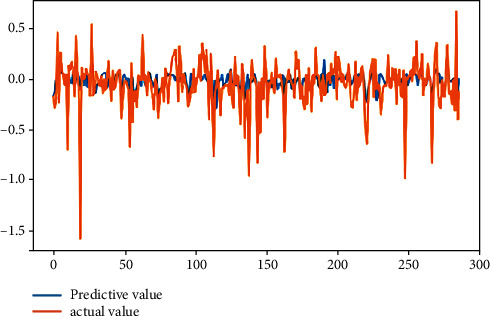
Fitting effect diagram of real earnings' management.

By setting the parameters into the gradient boosting decision tree, the accrued earnings' management *R*^2^ is calculated to be 0.3741322 with MSE 0.0102545, and the true earnings' management *R*^2^ is 0.2579867 with MSE 0.0505632. It can be seen that the fitting degree of the two is high and their fitting effect is good. The fitting effect of Gradient Boosting Decision Tree can be seen from Figures 9 and 10.

## 4. Applications

**Figure 11:**
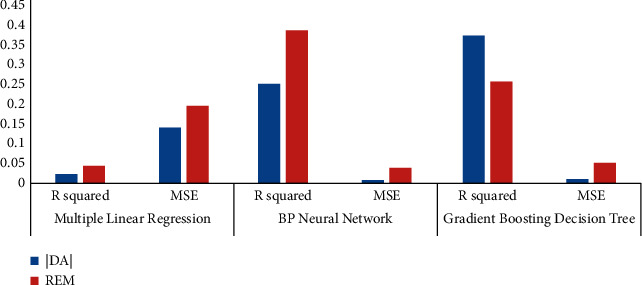
The comparison of prediction ability of multiple models.

As shown above, all three models can forecast GEM earnings' management, but it can be concluded by comparison that there are some limitations for the traditional multiple linear regression model on the analysis of the small- and medium-sized plate; that is to say, the fitting effect is poorer and cannot predict the degree of earnings management very well. However, the prediction ability of the BPNN model and the GBDT model is obviously superior to the regression model. There is a straightforward explanation: it is not a simple linear relationship between elements of corporate governance structure and earnings' management in the real world, so the rest two kinds of models are more suitable for the study and the simulation result shows better. In addition, in the comparison of the neural network model and the gradient boosting decision tree model, we can find that, in the study of accrued earnings' management, the prediction effect of the gradient boosting decision tree is slightly higher, and the error is smaller, but in the prediction of real earnings' management, the neural network was slightly higher than the GBDT model. Therefore, in future research, we can choose a different model according to the different ways of earnings management. *R*^2^ and MSE of BPNN, GBDT, and MLR models are shown in Figure 11.

## 5. Conclusion

This study selects 2987 observation values of listed companies on China Growth Enterprise Market from October 30, 2011, to December 31, 2020, studies the relationship between corporate governance structure and earnings' management, respectively, by using multiple linear regression, the BPNN, and the gradient boosting decision tree, and makes a comparative analysis of the empirical research results of the three models. The results show that the latter two models have high goodness of fit and small error and are more suitable for different kinds of earnings' management, which opens up a new way for future research on the nonlinear relationship.

## Figures and Tables

**Table 1:** Descriptive statistics.

Variables	Mean	Std. dev.	Min	Max
|DA|	0.0787586	0.1422875	0	2.8347
REM	−0.0121829	0.2007963	−1.7549	2.8589
Share proportion of the largest shareholder	30.8848800	12.2357400	4.08	81.1
The proportion of state-owned shares	0.1820544	1.4169230	0	29.95
The legal person shareholding ratio	0.0705015	0.1497494	0	0.8216
The condition of one person taking the chairman of the board and the general manager	0.4338041	0.4957081	0	1
Proportion of the independent directors	38.5460900	5.5476830	20	60
Number of the directors	7.8595260	1.4069040	4	13
The shareholding ratio of members of the board of supervisors	0.0045202	0.0132673	0	0.154289
The company size	21.4498200	0.8055828	19.20553	25.11152
Return on the equity	0.0056593	0.8427084	−22.5441	0.749911
The asset-liability ratio	0.3297066	0.1821164	0.011054	1.685267
Total size of supervisors	3.0654080	0.3916420	0	6

**Table 2:** The results of linear regression analysis.

Variables	|DA|			REM		
	Coef.	*t*	*P* > *t*	Coef.	*t*	*P* > *t*
Share proportion of the largest shareholder	−0.00062	−2.39	0.017	−5.7*E* − 05	−0.16	0.874
The proportion of state-owned shares	−0.00024	−0.11	0.912	0.002611	0.87	0.383
The legal person shareholding ratio	0.024021	1.18	0.238	0.055345	1.95	0.051
The condition of one person taking the chairman of theboard and the general manager	0.003023	0.49	0.621	0.006022	0.71	0.48
Proportion of the independent directors	−0.00057	−0.79	0.427	−0.00058	−0.57	0.567
Number of the directors	−0.00093	−0.32	0.748	0.001601	0.4	0.691
The shareholding ratio of members of the board of supervisors	−0.2949	−1.27	0.204	−0.13363	−0.41	0.68
The company size	−0.00554	−1.33	0.184	0.0047	0.81	0.418
Return on the equity	−0.01501	−4.15	0	−0.00502	−0.99	0.32
The asset-liability ratio	0.060572	3.17	0.002	0.215889	8.1	0
Total size of supervisors	0.01993	2.55	0.011	−0.00272	−0.25	0.803
_Cons	0.163024	1.66	0.097	−0.17102	−1.25	0.212

**Table 3:** The model parameter selection.

Names of parameter	Values	Names of parameter	Values
Learning_rate	0.1	Min_samples_split	2
*n*_estimators	80	Min_samples_leaf	1
Max_depth	3	Max_features	14

## Data Availability

The data that support the findings of this study are available from the corresponding author upon reasonable request.
